# Reconstruction of medial patellofemoral ligament with adductor magnus tendon for recurrent patellar dislocation in children: a retrospective comparative cohort study

**DOI:** 10.1186/s13018-023-04221-6

**Published:** 2023-09-27

**Authors:** Yuqi Wang, Yi Zhao, Xincheng Huang, Zhuolin Lei, Hong Cao

**Affiliations:** 1 Department of Orthopedic Surgery, Renmin Hospital, Hubei University of Medicine, Shiyan, 442000 Hubei People’s Republic of China; 2 Department of Plastic and Burn, Renmin Hospital, Hubei University of Medicine, Shiyan, Hubei People’s Republic of China

**Keywords:** Recurrent patellar dislocation, Children, Adductor magnus tendon, Medial patellofemoral ligament, PEEK suture anchors

## Abstract

**Background:**

The purpose of current retrospective study was to explore the outcomes of using the adductor magnus tendon to reconstruct the medial patellofemoral ligament in the treatment of recurrent patellar dislocation in children.

**Method:**

Thirty-two children with recurrent patellar dislocation were selected. Sixteen cases in the conservative group, seven males and nine females, with an average age of 11.81 ± 1.28 years; sixteen cases in the surgical group, eight males and eight females, with an average age of 11.56 ± 1.15 years. All patients had no surgery history. The IS index (> 1.2), *Q* angle (> 20°) and tibial tubercle–femoral trochlear groove (TT–TG) distance (> 20 mm) were measured by X-ray and MRI. The conservative group was treated with closed reduction and a brace, and the surgical group received surgical treatment. Two years after surgery, congruence angle (CA) (− 6° to 6°) and lateral patellofemoral angle (LPFA) (7.7°–18.7°) were measured by X-ray image and all children were evaluated based on Kujala and Lysholm scores. The re-dislocation rate was recorded. Analysis was performed by t test and chi-square with the statistical SPSS software. *P* < 0.05 was considered a statistically significant difference. Furthermore, we measured the length (mm) of the adductor tendon and MPFL in three knee cadaveric specimens, and also observed the positional relationship between the two structures.

**Result:**

There were no significant differences in sex, age, injury site between groups (*P* > 0.05). Patients in the two groups were followed up for 2 years in average. Among the 16 cases in the conservative group, 7 cases (43.75%) had recurrence of patellar dislocation, while none of recurrence in the surgical group (*P* < 0.05). The Lysholm score of the surgical group (94.63 ± 8.99) was significantly better than that of the conservative group (79.31 ± 18.90), and the Kujala score of the surgery group (95.25 ± 10.32) was also significantly better than that of the conservative group (77.06° ± 14.34°) (*P* < 0.05). The CA and LPFA of the two groups of patients after treatment were significantly recovered. The CA (− 5.81° ± 7.90°) in the surgical group was significantly better than that in the conservative group (20.94° ± 8.21°), and the LPFA (6.44° ± 3.22°) was also significantly better than that in the conservative group (− 9.18 ± 11.08), and the difference is statistically significant (*P* < 0.05). We found it through autopsy that adductor magnus tendon was 124.33 ± 1.53 mm long, MPFL was 48.67 ± 2.08 mm, and the femoral insertion of the adductor magnus tendon was adjacent to the MPFL femoral insertion.

**Conclusion:**

Reconstruction of Medial patellofemoral ligament with the adductor magnus tendon, fixing with PEEK suture anchors on the patellar side, can achieve satisfactory results in the treatment of children with recurrent patellar dislocation. Compared with conservative treatment, the rate of recurrence is lower and the stability of the patella is better.

## Introduction

Recurrent patellar dislocation is one of the common knee injuries and is more likely to occur in children and active populations [1]. Dislocations can lead to articular cartilage injuries, osteochondral fractures, recurrent instability, pain, decreased activity and patellofemoral arthritis [2]. It is estimated that about 44% of children will experience recurrent dislocations after the initial dislocation [3]. In the past, it was considered that recurrent patellar dislocation in children could be managed conservatively. However, a study of conservative treatment involving 470 patellar dislocations [4] showed that 30.9% of patients had recurrent dislocations during a mean follow-up of 3 years. There were also patients with persistent patellofemoral pain and subluxation, leading to unsatisfactory follow-up outcomes [5].

Therefore, more and more studies focus on surgical treatment. Soft tissue surgery to reconstruct the medial patellofemoral ligament (MPFL) is an important option for children with incomplete maturation of the skeletal system [6]. Studies have shown that the MPFL contributes 50% of the lateral restraining force to maintain patella stability [7]. Almost all children with recurrent patellar dislocation have MPFL injuries [8]. There are many ways to reconstruct an MPFL. The main difference between current surgical approaches is the choice of graft. In the last century, Avikainen et al. [9] described a study involving 14 patients. They reconstructed the MPFL by using adductor magnus tendon. Twelve patients had good outcomes at 7 years of follow-up after surgery, but the lack of a control group was a limitation of the study. In addition, we were accustomed to using metal rivets or bone channels for patellar fixation of tendons grafts. But the use of metal rivets increased the risk of metallic foreign body, and the bone tunnels run the risk of blowout in skeletally immature individuals. Therefore, we chose PEEK suture anchors which had good biocompatibility, chemical inertness and a similar modulus of elasticity to human bone.

The purpose of this study was to investigate the outcome of MPFL reconstruction using the adductor magnus tendon and PEEK suture anchors to maintain patellar stability. To observe the postoperative functional recovery effect and dislocation recurrence of the children when combined with lateral retinaculum release.

## Materials and methods

### Patient selection criteria

We grouped patients according to the treatment they received. The surgical group included the patients who received surgical treatment, and the conservative group included the patients who received conservative treatment. When patients initially choose treatment methods, they choose independently after explanations from doctors. After calculating the sample size, we randomly selected 16 patients from the two groups, a total of 32 patients, for data collection and analysis. All children have received treatment before this study, and we will not give intervention again. Inclusion criteria: ① Patellar instability was assessed by X-ray and MRI: high-riding patella (IS index > 1.2), *Q* angle > 20°, TT–TG distance > 20 mm; ② The children had a history of at least one incident of patellar dislocation before, and the same side was dislocated again. ③ The children have no history of knee surgery; ④ Family members and the children agreed to surgical treatment or required conservative treatment and signed their informed consent. Exclusion criteria: ① Presence of cerebral palsy or congenital neurogenic disease; ② Initial traumatic, congenital, fixed and neurogenic patellar dislocation; ③ Open knee injury; ④ No medial patellofemoral ligament injury.

All patients in the operative group were operated by the same orthopedic surgeon. A team composed of one radiologist and two orthopedic surgeons counted the basic data and imaging data (IS index, *Q* angle, TT–TG distance, CA and LPFA). They also followed up patients to count the recurrence rate of patellar dislocation and completed Kujala and Lysholm scores. This retrospective study was approved by the ethics committee of dangdi the institution, including: the use of patient medical record data, the use of anatomical specimens, etc.

### General information

The conservative group consisted of 7 males and 9 females, aged 10–14 years, with an average of 11.81 ± 1.28 years. There were 7 sprains, 7 falls and 2 impact injuries. There were 7 cases of left knee injury, 9 cases of right knee injury, and 4 cases of cartilage injury. The surgical group consisted of 8 males and 8 females, aged 10–14 years, with a mean of 11.56 ± 1.15 years. There were 7 cases of sprain, 6 cases of a fall, and 3 cases of impact injury. There were 9 cases of left knee injury, 7 cases of right knee injury, and 4 cases of cartilage injury. There was no significant difference in demographics between the two groups (*P* > 0.05). The preoperative IS index, *Q* angle, TT–TG distance, Congruence angle and lateral patellofemoral angle of the two groups of patients are shown in Table 1.

**Table 1:** Comparison of general data of the two groups of patients

	Conservative group	Surgical group	*t* value/*χ*^2^ value	*P* value
Gender (male/female)	7/9	8/8	0.125	0.723
Age (years)	11.81 ± 1.28	11.56 ± 1.15	1.292	0.216
IS index > 1.2 (n)	11	12	0.155	0.694
*Q* angle (°)	30.56 ± 4.76	30.13 ± 5.19	0.22	0.836
TT–TG distance	24.56 ± 2.58	25.00 ± 2.50	1.051	0.31
Post-injury CA (°)	63.69 ± 11.52	61.75 ± 9.77	0.596	0.578
Post-injury LPFA (°)	− 58.31 ± 9.06	− 59.88 ± 8.61	0.485	0.634

### Treatment methods

**Fig. 1:**
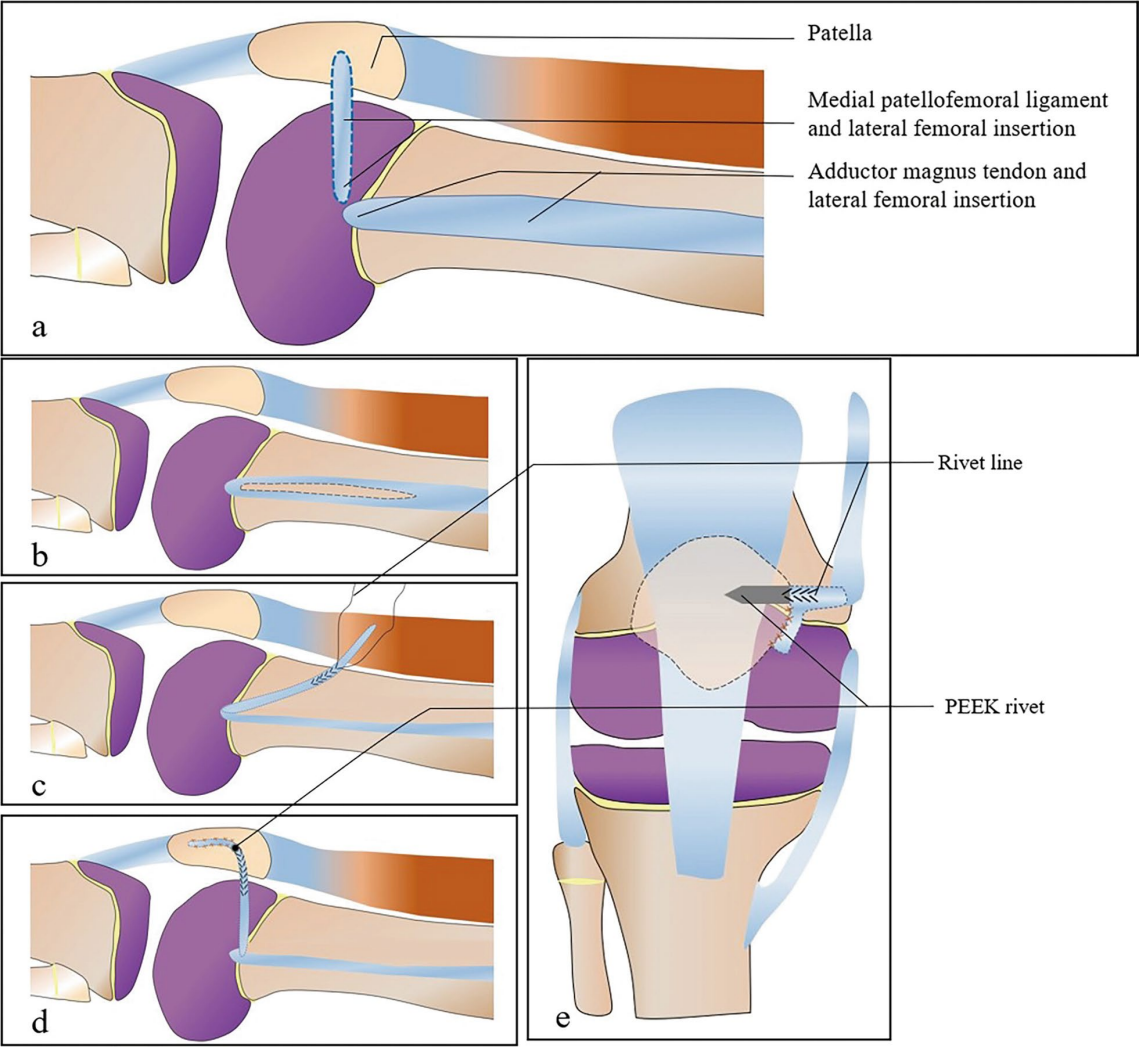
Schematic diagram of the surgical operation: in normal anatomy, the femoral side insertion of the medial patellofemoral ligament is adjacent to the femoral side insertion of the adductor magnus tendon (**a**). Intraoperative dissection was performed to expose the adductor magnus tendon and cut it from the middle to divide the adductor magnus tendon into two bundles (**b**). The end of the tendon was braided with rivet tail wire (**c**), the rivet and tail wire were finally fixed to the medial side of the patella, and the excess tendon was sutured to the patella in an “L” shape (**d**, **e**)

**Fig. 2:**
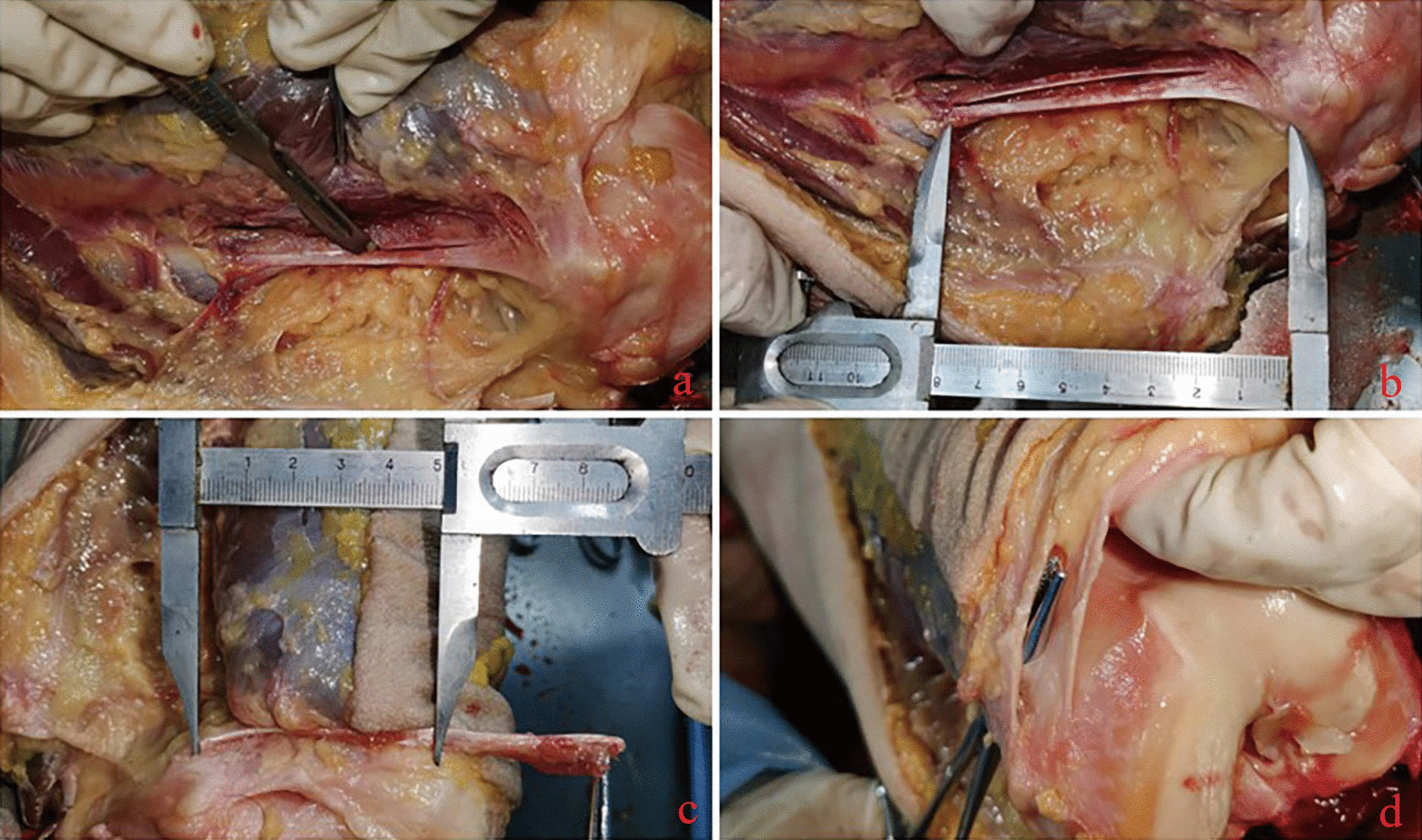
Anatomical diagram of ligament reconstruction: when obtaining the transplanted tendon, the adductor magnus tendon was split longitudinally from the middle (**a**), and the half tendon was cut at the proximal end to obtain an approximately 8 cm long tendon. The distance between the insertion point of the femur and the medial border of the patella in children was approximately 5 cm (**b**, **c**). The acquired adductor magnus tendon was passed through the medial subcutaneous tissue channel during tendon reconstruction to avoid further damage to the anatomy of the child’s knee (**d**)

**Fig. 3:**
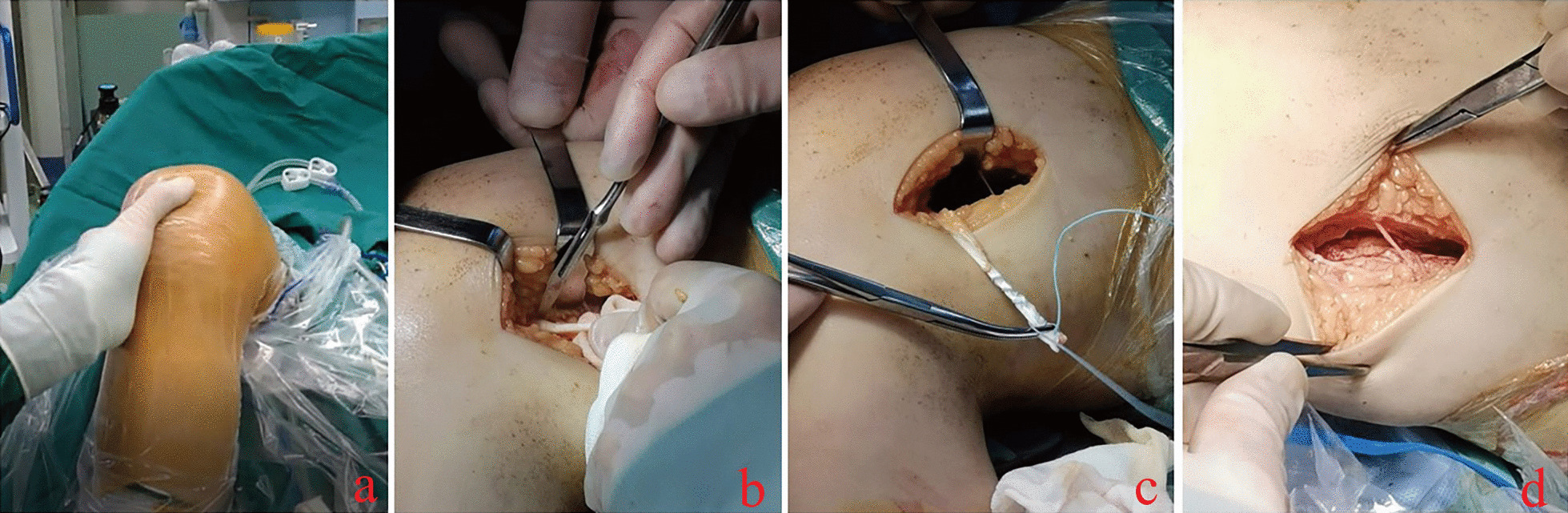
Intraoperative findings: During the operation, the trajectory of the patella was evaluated before the arthroscopic operation, and the patellar extrapolation experiment was performed. This child with complete dislocation had a feeling of emptiness in the trochlea of the femur, and the patella was significantly moved outward (**a**). Intraoperatively, the graft tendon was removed as much as possible to the insertion point, but the insertion site was not damaged (**b**). The tendon was braided with a rivet tail (**c**), and the excess tail was cut for an “L”-shaped suture to free the tendon. The tendon of the adductor magnus was preserved intraoperatively to maintain function (**d**)

The conservative group was treated with closed reduction and a brace for 4 weeks, and then the brace was removed to gradually guide the movement of the affected limb during follow-up. Patients in the surgical group underwent general anesthesia. The schematic diagram of the operation (Fig. 1), the anatomical diagram (Fig. 2) and the intraoperative findings (Fig. 3) are presented as follows:

① Arthroscopy of the knee joint
  Assessment of the patellar stability is conducted under general anesthesia prior to commence of surgical procedure (Fig. 3a). Then we perform a knee joint examination assisted by knee arthroscopy [10]. We observe the ligament and cartilage damage in the knee joint. If cartilage damage is present, this should be dealt with prior to MPFL reconstruction [11].
② Acquisition of the adductor magnus tendon
  Skin incision, about 5 cm, is made at the superomedial aspect of the knee joint to expose the adductor magnus tendon and make a longitudinal incision through the center of the tendon (Figs. 1b, 2a). We severed half of the adductor magnus tendon, taking care to protect the nerves and blood vessels at the adductor hiatus. The surgeon stripped the tendon close to the femoral insertion, while the distal insertion on the femoral condyle is retained (Fig. 3b). The lateral femoral insertion of the tendon was not cut, and an approximately 8 cm long tendon was incised (Fig. 2b, c).
③ Reconstruction of the medial patellofemoral ligament
  We pass the adductor magnus tendon through the extracapsular soft tissue channel (Fig. 2d), and use the Ultra high molecular weight polyethylene (UHMWPE) suture of PEEK suture anchors to weave the tendon (Figs. 1c, 3c) and initially fix it onto the medial border of the patella. The knee joint is fully movable in the anterior range, and the fixation position and length of the graft are determined according to the trajectory and stability of the patella. The patella should be mobile about 7–9 mm. The transplanted tendon should be able to play a “rein” role, and the patella should be stably fixed in the trochlear groove of the femur without any impaction or impingement when the knee is flexed [12]. After final confirmation, with the knee flexed at 30°, we inserted the PEEK suture anchors into the upper part of the medial border of the patella, and sutured the excess tendon to the medial border of the patella. The graft tendon is fixed to the medial side of the patella in an “L” shape to increase the stability of the fixation (Fig. 1d, e). We retained half of the adductor magnus tendon to maintain function (Fig. 3d).
④ Review the stability of the patella
  The knee joint was moved again, and the movement trajectory of the patella and the relaxation and contraction of the transplanted tendon were observed. Intraoperatively, we performed fluoroscopic knee radiographs to confirm patella height and trajectory.

Special Note: The surgical methods studied in this study were still used in clinical treatment when the manuscript was written. Although this study is a retrospective analysis, we can still describe the operation process in detail. Figure 3 was collected before the study, which was completely consistent with the surgical operation received by the research target. The collection and use of the images were approved by the ethics committee and the consent of parents of children.

### Postoperative treatment

Both groups of patients were treated with braces for 4 weeks. The quadriceps muscle was actively exercised during immobilization to enhance muscle strength and prevent muscle atrophy. After 4 weeks, the affected limbs were active and passively exercised, with partial weight-bearing after 6 weeks, and full weight-bearing after 3 months. During the functional recovery period, strenuous activities such as uphill and downhill, stair climbing, running and jumping were prohibited. Follow-up was continued for 2 years. During the follow-up period, the children were closely assessed for the recurrence of dislocation. The X-ray was reviewed at the last follow-up to observe the position of the patella, and CA and LPFA were estimated.

### Statistical methods

The data collected in the study were analyzed using SPSS 25.0 software. The count data, such as each score and angle, were expressed as the mean ± standard deviation, and a t test was performed. The measurement data, such as sex, location and recurrence, were tested by the *χ*2 test, and the difference was statistically significant at *P* < 0.05. We used the Walters normal approximation method to approximate the Fisher exact probability method (*α* = 0.05, 1−*β* = 0.90). The results showed that when the sample size of both groups was 12 cases, the total sample size was 24, with power > 0.90.

## Results

### Postoperative follow-up efficacy

There were no significant differences in sex, age, injury site, IS index, *Q* angle, TT–TG distance, preoperative CA and LPFA between groups (*P* > 0.05) (Table 1). The mean follow-up time of patients in the conservative group was 25.69 ± 1.44 months, and that in the surgical group was 25.81 ± 1.42 months. There were not early or late infections, arthrofibrosis, implant-related failures in the surgical group. Among the 16 cases in the conservative group, 7 cases (43.75%) had recurrence of patellar dislocation, while none of recurrence in the surgical group (*P* < 0.05) (Table 2). The Lysholm score of the surgical group (94.63 ± 8.99) was significantly better than that of the conservative group (79.31 ± 18.90), and the Kujala score of the surgery group (95.25 ± 10.32) was also significantly better than that of the conservative group (77.06° ± 14.34°) (*P* < 0.05) (Table 2). The CA and LPFA of the two groups of patients after treatment were significantly improved. The CA (− 5.81° ± 7.90°) in the surgical group was significantly better than that in the conservative group (20.94° ± 8.21°), and the LPFA (6.44° ± 3.22°) was also significantly better than that in the conservative group (− 9.18 ± 11.08), and the difference was statistically significant (*P* < 0.05) (Table 2).

**Table 2:** Comparison of the follow-up of the two groups of patients

	Conservative group	Surgical group	*t* value/*χ*^2^ value	*P* value
Follow-up time(month)	25.69 ± 1.44	25.81 ± 1.42	− 0.264	0.795
Number of recurrent dislocations	7	0	8.96	0.003
Follow-up CA (°)	20.94 ± 8.21	− 5.81 ± 7.90	7.778	< 0.001
Follow-up LPFA (°)	− 9.18 ± 11.08	6.44 ± 3.22	− 4.755	< 0.001
Follow-up Lysholm Score	79.31 ± 19.90	94.63 ± 8.99	− 3.003	0.009
Follow-up Kujala score	77.06 ± 14.34	95.25 ± 10.32	− 6.171	< 0.001

### Observations on cadaver specimens

**Fig. 4:**
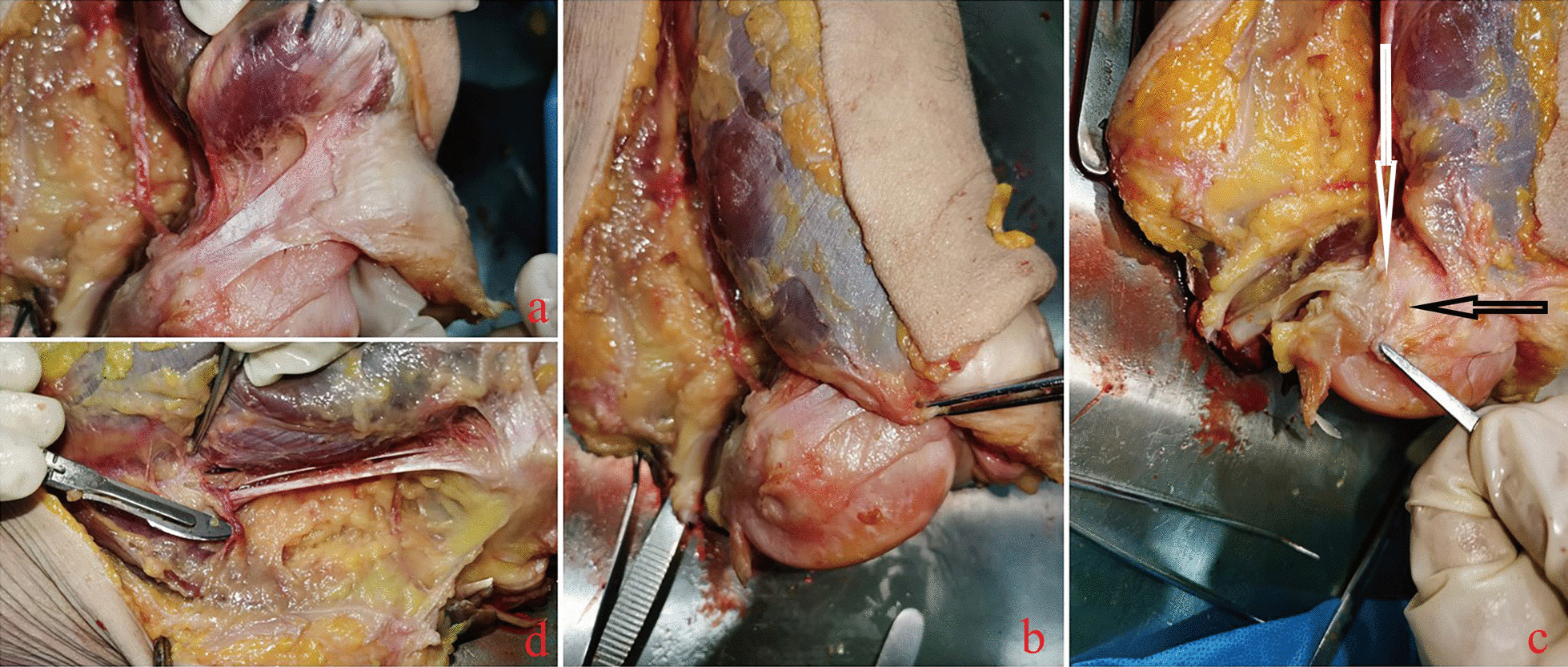
Most of the medial patellofemoral ligament is a thin membrane structure (**a**), which is very vulnerable to injury. Some studies have used it as part of the medial retinaculum of the knee joint. Normally, the medial patellofemoral ligament runs under the internal oblique muscle, emerges from the soft tissue distal to the internal oblique muscle, and attaches to the medial border of the patella (**b**). The adductor magnus tendon insertion (white arrow in **c**) has been found by knee autopsy to be adjacent to the medial patellofemoral ligament insertion (black arrow in **c**). Special attention should be given to protecting important structures, such as the proximal vessels, during tendon dissociation (**d**)

In this study, three cadaveric knee joint specimens were used to analyze the anatomical feasibility of the adductor magnus tendon before modified reconstruction. The anatomical feasibility is as follows: ① The adductor magnus tendon can be freed up to the proximal transition when reconstructing the MPFL, where the length from the insertion point of the femur is approximately 124.33 ± 1.53 mm, and the length of the medial patellofemoral ligament (axial length) is approximately 48.67 ± 2.08 mm; ② The femoral insertion of the adductor magnus tendon is adjacent to the MPFL femoral insertion (Fig. 4c). Retaining the adductor magnus insertion during reconstruction can mimic the physiological function of the MPFL to the greatest extent.

## Discussion

Our study explored the surgical outcomes of MPFL reconstruction using the adductor magnus tendon and PEEK suture anchors. There was no patellar dislocation during follow-up in the surgery group, this was an acceptable situation. But the absence of recurrence of dislocation might be associated with shorter follow-up. In the original study by Avikainen et al. [9], all patients were followed up for 7 years, and 12 patients achieved good results. Similarly, Sillanpää et al. [13] used the adductor magnus tendon for MPFL reconstruction and reported recurrent patellar dislocation in only 1 patient (7%) during 10-year follow-up. This study supported use of adductor tendon for MPFL reconstruction in a small series of 47 patients. Our study also applied PEEK suture anchors to MPFL reconstruction. Compared with other fixation methods, PEEK material has better biocompatibility and more stable chemical inertness. And it has similar modulus of elasticity to that of human bone. This avoids “stress shielding” effects. When the modulus of the implant material is higher than that of the bone tissue, the implant structure will support more stress, and the bone tissue without stress stimulation will be tissue degradation, which is manifested in the decrease of bone density and osteoporosis. It is not conducive to the later growth and rehabilitation of bone tissue [14–16].

There are many surgical modalities to reconstruct MPFL described in the study [17–19]. One advantage of reconstructing (not repairing) is replacing torn or stretched ligaments with grafts containing collagen fibers, rather than stretching, tightening, and immobilizing damaged tissue [20]. Anatomy shows that MPFL is a flat membrane structure (Fig. 4a, b). This special anatomical structure makes it easy to be damaged by external forces. The main difference in current surgical approaches is the choice of graft. Alm et al. [21] used gracilis tendon to reconstruct MPFL for patellar dislocation in 30 knees. Follow-up showed that 87% of patients had excellent postoperative scores and the patella was stable, but there were still 4 recurrent dislocations (13%). Kang et al. [22] used the semitendinosus tendon for reconstruction, and there was no recurrence of dislocation at a 2-year follow-up. However, the use of gracilis tendon or semitendinosus tendon to reconstruct MPFL requires femoral fixation, and its impact on subsequent growth and development in children is unclear. At present, there is no study comparing the clinical effect of each autologous tendon graft, and according to the existing reports, each graft has a good effect on avoiding the recurrence of dislocation. But for growing children, recurrence of the dislocation is not the only factor to consider. It cannot be ignored that gracilis tendon [23], semitendinosus tendon [24] and other tendons [25, 26] (hamstring tendon, etc.) need to be completely freed and then double-fixed at the femoral and patellar. The freed tendons cannot continue to perform their original functions [27].

In order to minimize the trauma to the child during surgery, Sillanpää et al. [14] proposed a reconstruction method that preserves the lateral femoral insertion of the adductor magnus tendon. We used the same surgical approach as Sillanpää, with a longitudinal split of the adductor magnus tendon, leaving half of the adductor magnus tendon to continue functioning. Surgical feasibility: In a cadaveric study, Milinkovic et al. [28] found that the average length of the adductor magnus tendon was 12.6 ± 1.5 cm and the average distance from the insertion point of the adductor tubercle to the adductor hiatus was 10.8 ± 1.3 cm which exceeded the mean expected length of the graft (7.5 ± 0.5 cm) by 3.3 ± 0.7 cm. At the same time, the cadaveric study also found that the adductor magnus tendon was adjacent to the lateral femoral insertion of the MPFL (Fig. 4c), which provided the possibility of preserving the femoral insertion of the adductor magnus tendon while ensuring a fixed anatomical reconstruction [29]. This is similar to our cadaveric study.

Finally, the surgical approach used in this study avoided destruction of the femur during reconstruction by other tendons (rivet fixation on the femoral) or the anatomical difference from MPFL when performing reconstruction by bypassing the adductor hiatus. Of course, Anatomical risk factors must be taken into account when harvesting the adductor magnus tendon, the nerves and blood vessels surrounding the tendon, such as the descending geniculate artery and its branches, must be carefully protected (Fig. 4d). The function of the adductor magnus can still be maintained, and the damage to the anatomical structure can be avoided to the greatest extent. PEEK suture anchor was used to fix the transplanted tendon and sutured the excess tendon to the medial border of the patella in an “L” shape to increase the contact area, which could maximize the fixation strength and avoided the risk of metal screws.

The follow-up time of this study was short, and follow-up observation would be continued.

## Conclusion

Reconstruction of Medial patellofemoral ligament with the adductor magnus tendon, fixing with PEEK suture anchors on the patellar side, can achieve satisfactory results in the treatment of children with recurrent patellar dislocation. Compared with conservative treatment, the rate of recurrence is lower and the stability of the patella is better. But the follow-up time of this study was short, and follow-up observation would be continued.

## Data Availability

All data generated or analyzed during this study are included in this published article.
